# Partitioning beta diversity in a tropical karst seasonal rainforest in Southern China

**DOI:** 10.1038/s41598-018-35410-7

**Published:** 2018-11-27

**Authors:** Yili Guo, Wusheng Xiang, Bin Wang, Dongxing Li, Azim U. Mallik, Han Y. H. Chen, Fuzhao Huang, Tao Ding, Shujun Wen, Shuhua Lu, Xiankun Li

**Affiliations:** 1 Guangxi Key Laboratory of Plant Conservation and Restoration Ecology in Karst Terrain, Guangxi Institute of Botany, Guangxi Zhuang Autonomous Region and Chinese Academy of Sciences, Guilin, 541006 China; 2 Guangxi Youyiguan Forest Ecosystem National Research Station, Pingxiang, 532600 China; 3 Faculty of Natural Resources Management, Lakehead University, Thunder Bay, Ontario P7B 5E1 Canada; 4 Faculty of Biology, Lakehead University, Thunder Bay, Ontario P7B 5E1 Canada

## Abstract

Both deterministic and stochastic processes have been linked to forest community assembly; however, their contribution to beta diversity has not been properly explored, and no studies to date have investigated their impacts on sparse depleted soils in forests that contain widespread exposed limestone karst. We found that the pairwise differences in species composition between quadrates was determined by a balanced variation in abundance, whereby the individuals of some species at one site were substituted by an equivalent number of individuals of different species at another site. Both the total beta diversity and its balanced variation in abundance declined with increasing sampling grain size. Our research indicated that environmental differences exert a strong influence on beta diversity, particularly total beta diversity and its balanced abundance variation in larger grain sizes. It was evident that deterministic and stochastic processes worked together, and that deterministic processes were more important than stochastic processes in the regulation of beta diversity in this heterogeneous tropical karst seasonal rainforest of Southern China. However, in future research a functional trait based approach will be required to tease out the relative degree of deterministic and stochastic processes toward an assessment of the temporal changes in species composition.

## Introduction

Patterns of site-to-site variations in community composition (beta diversity) can provide fundamental insights into the processes that create and maintain species diversity^1,2^. Community dissimilarity is determined by two distinct processes: species replacement (also called turnover) and richness differences or nestedness (species gain and loss)^3^. These two processes can operate in many different ways, which influence species distribution across spatial and temporal scales toward the ultimate formation of complex patterns of community dissimilarity^4^. However, the widely used broad scale measures of compositional dissimilarity are not able to reveal the processes and underlying mechanisms that drive community assembly^5^. Despite its apparent simplicity, dissimilarity is a concept that has proven to be elusive. This is because both assemblage composition and differences therein may be defined in several ways^6^. Even once the composition definition is set, an array of dissimilarity definitions are possible^7^, with each accounting for different facets of dissimilarity.

With methodological advances, some of these limitations have been overcome. Most recently, Baselga^6,8^ and Podani, *et al*.^9^ provided two alternative frameworks to separate beta diversity into two components of abundance-based assemblage dissimilarity. Typically, the two frameworks, with different conceptual and mathematical backgrounds, are referred to as the Baselga and Podani families^10^. The Baselga family separates beta diversity into two components: (i) balanced variation in abundance, whereby the individuals of some species at one site are substituted by the same number of individuals of different species at another site, and (ii) abundance gradients, whereby some individuals are lost from one site or the other^6,8^. The Podani family also involves two fractions, where one is derived from differences between total abundance, and the other from differences due to abundance replacement^9^. Both families contain indices for presence/absence based, and abundance based, dissimilarity. Further, there are many indices (e.g., the Jaccard and Sørensen index) that decompose dissimilarity into replacement, richness or abundance, and nestedness components. Subsequent contributions have shown that either methodological approach may be applied to both abundance-based versions of Jaccard and Sørensen indices^10^.

Although the debate regarding the two families persists as to which is the “standard” method of beta diversity partitioning^11–13^, the forms available for both presence/absence based and abundance based data are useful, as these different data types allow researchers to elucidate different aspects of ecosystem functionality or biogeographic processes^10^. With the methodological advantages of these partitioning frameworks, our understanding of the cryptic processes of diversity patterns in some taxa has improved over a broad range of spatial scales^14^. Nonetheless, almost all previous studies have been conducted across broad continental scales, and via the mechanisms behind variation in animal or algae species composition with presence-absence-based measures of dissimilarity. To date, few studies on the partitioning of beta diversity have been conducted in forest ecosystems with novel abundance-based metrics, one being from Sfair, *et al*.^15^. This novel partition may be useful toward the assessment of biodiversity patterns and for exploring the mechanistic underpinnings of these patterns, in that substitution and loss of individuals may derive from completely different processes^6^.

More recently, some authors have argued that stochastic processes (e.g., random birth and death, dispersal, speciation, and stochastic extinction) are sufficient to explain beta diversity patterns, even under the simplified assumption of no ecological differentiation between species^16,17^. Others have suggested that species specific niche differences are important, and therefore deterministic processes are critical to assemble species diversity toward a deterministic state^18^. For instance, environmental filtering of deterministic processes refers to abiotic factors that prevent the establishment, or persistence, of species in particular locations^19^. From this perspective, the ambient environment is seen as a selective force, which culls species that are unable to tolerate conditions at a particular site. These views differ from the emphasis given to deterministic vs. stochastic processes (i.e., niche vs. neutral processes); however, they are not mutually exclusive. Deterministic and stochastic processes jointly drive community assembly and meta-community dynamics, but it is difficult to quantify the relative importance of each process in natural vegetation, as their relative importance might vary with spatial scale^20,21^ and the quality or quantity of environmental data^22^.

Karst landscapes comprise about 15–20% of the Earth’s ice-free land surface; however, the basic study of this ecosystem lags far behind most other ecosystems. Karst ecosystems are sedimentary rock outcrops that consist primarily of calcium carbonate. Over millions of years, the softer sediments that covered the karsts have been degraded by mechanical and chemical weathering. This process typically produces “tower” and “cockpit” (*alias* Fengcong-Depression) karst formations in the tropics^23^. The strongly irregular geomorphology and good drainage of the limestone substrate, with many underground caves and cavities, result in more variegated vegetation. Therefore, karst forests are isolated habitats, and yet many floral taxa show aggregation patterns, possibly because of limited dispersal distance or narrow ecological niches^24^. Further, distinct species assemblages exist between different habitats, and most of the karst tree species exhibit consistent associations with a single habitat throughout their lifespans^25^. However, little is known regarding the mechanisms that drive tree species assembly in karst forests with pronounced topographical variation.

For this study, we focused on the spatial and temporal changes in abundance-based partitioned beta diversity and discerned the relative importance of the stochastic and deterministic processes that are linked to beta diversity and its components, in a long-term forest plot in a northern tropical karst seasonal rainforest of Southern China. We sought to address three questions: (1) How does partitioned beta diversity change along spatial and temporal scales? (2) How do the environmental drivers of the plot affect partitioned beta diversity? (3) To what extent is the partitioned beta diversity of a tropical karst rainforest explained by measured environmental variables, and by fitted spatial predictors? To address these issues, we initially investigated the dynamics of species assemblage between two censuses over a five year interval. Secondly, we quantified the spatial and temporal changes in the partitioned beta diversity components. Thirdly, we assessed whether the related topographic environmental drivers had significant effects on beta diversity dissimilarities. Finally, we tested the relative importance of stochastic and deterministic processes linked to beta diversity dissimilarities. Due to the diverse heterogeneous topographies of karst ecosystems, we hypothesised that stochastic and deterministic progress contributed jointly to the maintenance of beta diversity; however, deterministic processes such as environmental filtering would be more important than stochastic processes in the structuring of species assemblages in this type of forest.

## Results

### Dynamics of species assemblages between two censuses

**Figure 1:**
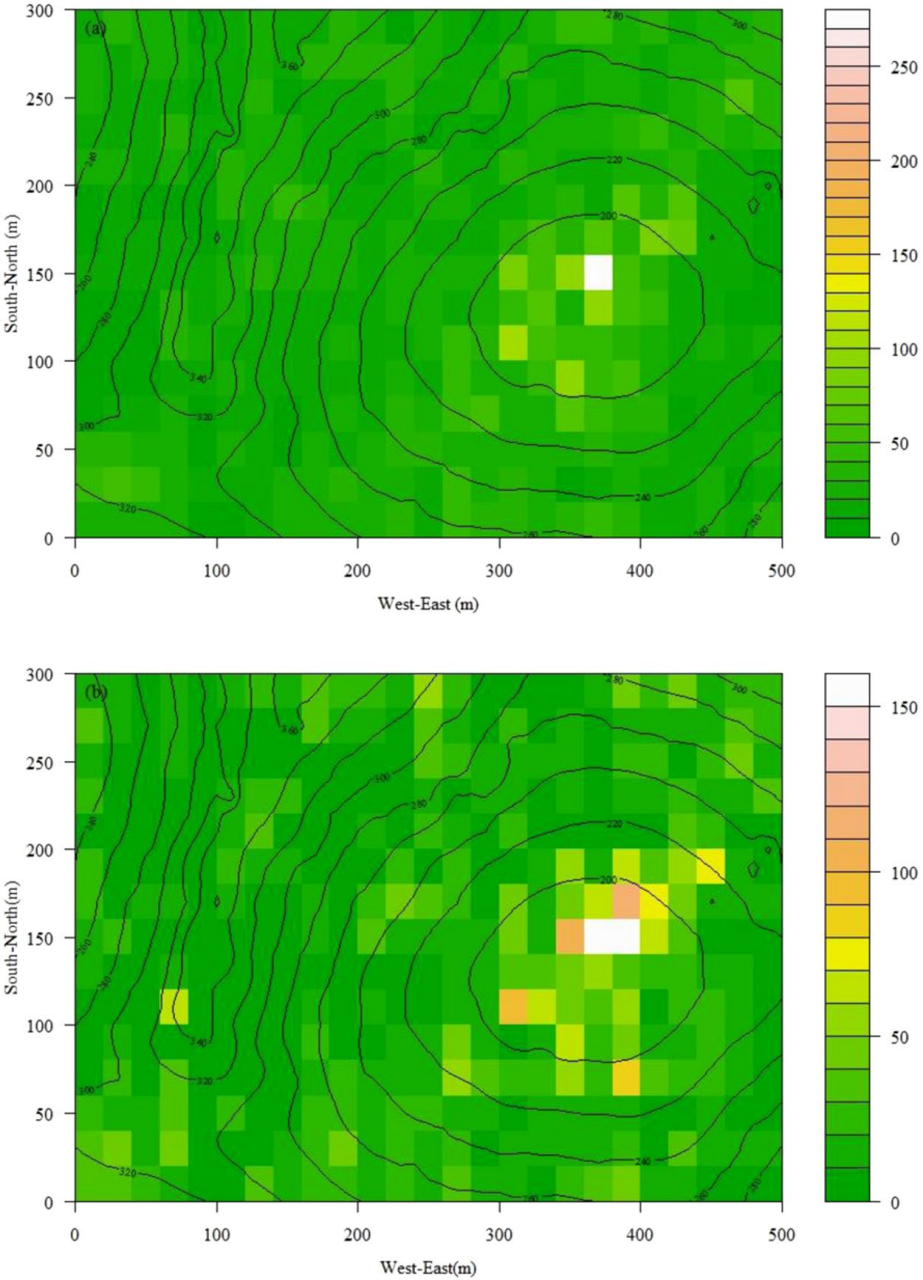
Contour map with 20 m intervals and the spatial pattern of dead individuals (**a**) and new individuals (**b**) for the 20 × 20 m cell between the 2011 and 2016 census in the 15-ha Nonggang plot.

Between the first and second census, a total of 9,791 individuals belonging to 193 species died, while only 3,893 new individuals belonging to 162 species appeared. Both the new and expired individuals appeared primarily in low-lying habitats, such as seasonally flooded depressions, peripheral depressions, and their adjacent areas; however, with little change of abundance in higher habitats (20 × 20 m cell size for example, Fig. 1). Two species (*Callicarpa cathayana* and *Dendrolobium triangulare*) with one individual each in the 2011 census died, with no new individuals added in the 2016 census.

Forty-one species with at least 100 individuals in the 2011 census died. Among them, *Sterculia monosperma* (1,802), *Ficus hispida* (1,792), *Cleistanthus sumatranus* (1,016), *Vitex kwangsiensis* (574), *Diplodiscus trichosperma* (545) and *Acalypha kerrii* (522) had the largest number of dead individuals. Only 17 species comprised of more than 100 individuals were newly added in the 2016 census. *Ficus hispida* (1,695), *Sterculia monosperma* (755), *Cleistanthus sumatranus* (455), *Vitex kwangsiensis* (224), *Pterospermum truncatolobatum* (221) and *Excentrodendron tonkinense* (205) had the largest number of new individuals. All of these species appeared to be distributed mainly in low-lying habitats.

### Dynamics of partitioned beta diversity components

**Figure 2:**
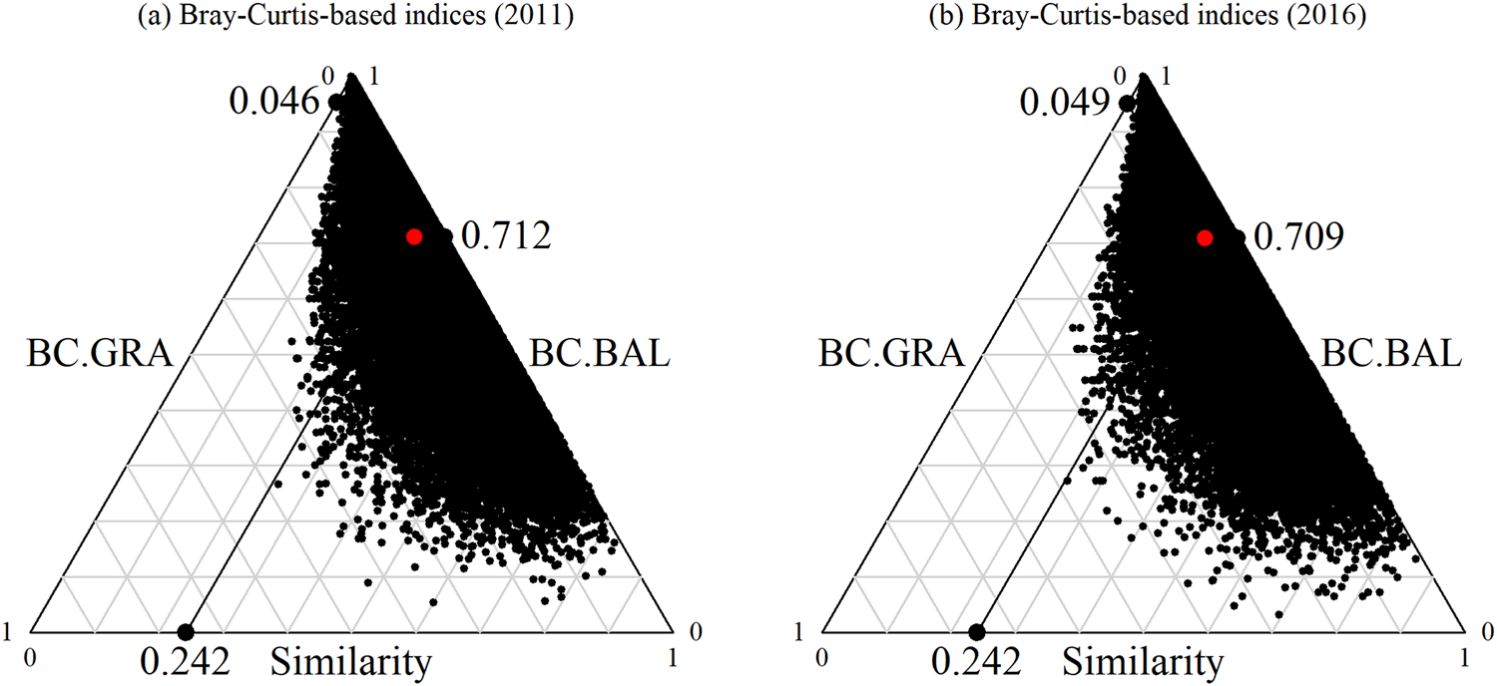
Triangular plots (simplices) of the relationships between the 70,125 pairwise inter-site values for the 20 × 20 m cell in the 15-ha Nnonggang plot. Each point (black dot) represents a pair of sites. Their positions were determined by a triplet of values from the Similarity = (1 − β_BC_), BC.BAL (balanced variation in abundance), BC.GRA (abundance gradients) matrices; each triplet sums to 1. The large central dot in each graph (red in the online version) is the centroid of the points; the larger dots (black in the online version) represent the mean values of the Similarity, BC.BAL, and BC.GRA components.

For all of the sampling grain cell sizes, high beta diversity was explained primarily by balanced variation in abundance (β_BC.BAL_), rather than difference in abundance gradients (β_BC.GRA_). Using the 20 × 20 m cell size as an example, the Bray-Curtis dissimilarity (β_BC_, mean = 0.758, SD = 0.158) was dominated by balanced variation in abundance (β_BC.BAL_, mean = 0.712, SD = 0.183), which implied that in any pairwise combination of quadrats between sites, an average of 71.2% of the individuals of a given species at one site were substituted by an equivalent number of individuals of a different species at another site. In contrast, the abundance gradient component (β_BC.GRA_, mean = 0.046, SD = 0.050) was much lower, which implied that no strong patterns of individuals of some species were lost from one site to another, in the 2011 census (Fig. 2a).

**Figure 3:**
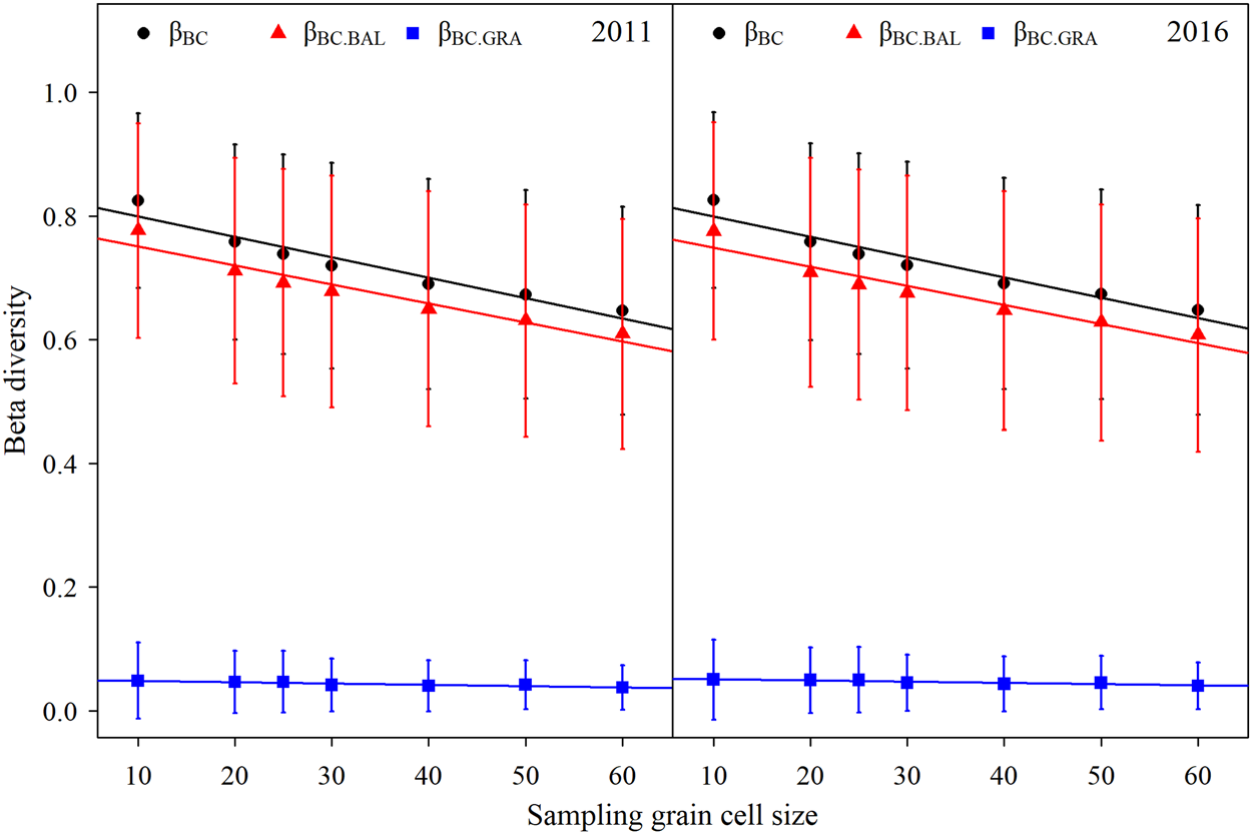
The trends of the different dissimilarity components of beta diversity along different sampling grain cell sizes (mean + SD) in the 15-ha Nnonggang plot. β_BC_: overall pairwise dissimilarity of Bray-Curtis; β_BC.BAL_: balanced variation in abundance; β_BC.GRA_: abundance gradients.

Furthermore, the proportions of the total, and the balanced variation in abundance were very high at a finer sampling grain cell size (10 × 10 m, up to 82.5% for total beta diversity, and 77.7% for balanced variation in abundance). However, this was systematically decreased with an increased sampling grain cell size (up to 64.9% for total beta diversity and 60.9% for balanced variation in abundance at 60 × 60 m). The proportion of negligible abundance gradients (only ~3–4%) also decreased with a larger sampling grain cell size (Fig. 3, Table 1). Although there was an obvious change of species abundance in certain habitats (Fig. 1), the amount of spatial assemblage heterogeneity, as measured by the pairwise inter-site dissimilarities in the plot, did not change significantly between the 2011 and 2016 census (Figs 2 and 3).

**Table 1:** Relationships between different components of beta diversity and the grain cell sample sizes using the Spearman method.

		2011		2016	
		*rho*	*p*-value	*rho*	*p*-value
Bray-Curtis dissimilarity	β_BC_	−1	0.0004	−1	0.0004
	β_BC.BAL_	−1	0.0004	−1	0.0004
	β_BC.GRA_	−0.8571	0.0238	−0.9285	0.0067

### Partitioned beta diversity determined by environmental drivers

We found identical effects of environmental drivers on overall beta diversity and its components, particularly at larger grain sizes. Environmental differences had significant positive effects on both total beta diversity and its balanced variation in abundance, with negative, albeit non-significant effects on their abundance gradients (Table 2).

**Table 2:** Effects of environmental drivers on pairwise dissimilarity of the Bray-Curtis (β_BC_) index and its partitioned components (balanced variation in abundance, β_BC.BAL_; abundance gradients β_BC.GRA_) for all grain cell sample sizes in the 15-ha Nonggang plot. Spearman correlation coefficients and significance levels resulting from the Mantel test are indicated.

Cell size	2011			2016		
	β_BC_	β_BC.BAL_	β_BC.GRA_	β_BC_	β_BC.BAL_	β_BC.GRA_
10	0.4229^***^	0.4175^***^	−0.2778	0.4229^***^	0.4175^***^	−0.301
20	0.5756^***^	0.5692^***^	−0.3184	0.5797^***^	0.5746^***^	−0.3321
25	0.6111^***^	0.6044^***^	−0.3146	0.6111^***^	0.6044^***^	−0.3741
30	0.6202^***^	0.6209^***^	−0.3594	0.6202^***^	0.6209^***^	−0.4183
40	0.6686^***^	0.6674^***^	−0.3788	0.6686^***^	0.6674^***^	−0.445
50	0.6538^***^	0.649^***^	−0.3345	0.6538^***^	0.649^***^	−0.4086
60	0.6533^***^	0.6425^***^	−0.3232	0.6533^***^	0.6425^***^	−0.3953

***P < 0.001.

Using the 20 × 20 m cell size as an example, overall beta diversity (β_BC_) was a significant positive (Spearman’s r = 0.5756, *P* < 0.001) function of environmental differences among pairwise inter-site stations, where this relationship was largely due to the positive trend (Spearman’s r = 0.5692, *P* < 0.001) in the balanced variation in abundance (β_BC.BAL_). However, there was a weak negative trend in the abundance gradients with environmental difference for the Bray–Curtis dissimilarity index (Spearman’s r = −0.3184, P = 0.2258) in 2011 (Table 3).

**Table 3:** Each of the topographic variable differences and the total difference effects on pairwise dissimilarity of the Bray-Curtis (β_BC_) index and its partitioned components (balanced variation in abundance, β_BC.BAL_; abundance gradients β_BC.GRA_) for the 20 × 20 m cell in the 15-ha Nonggang plot.

Environmental variable	2011			2016		
	β_BC_	β_BC.BAL_	β_BC.GRA_	β_BC_	β_BC.BAL_	β_BC.GRA_
ELE	0.6545^***^	0.6571^***^	−0.383	0.6557^***^	0.6562^***^	−0.384
SLO	0.4341^***^	0.4243^***^	−0.206	0.4395^***^	0.4319^***^	−0.2187
CON	0.3461^***^	0.3306^***^	−0.1765	0.348^***^	0.332^***^	−0.1845
TWI	0.2756^***^	0.2764^***^	−0.16	0.2837^***^	0.2848^***^	−0.1665
ACH	0.0379	0.0176	0.0759^*^	0.0421	0.0231	0.0619^*^
RBR	0.2493^***^	0.2464^***^	−0.1393	0.2447^***^	0.2426^***^	−0.142
SIN	0.0483^**^	0.0516^**^	−0.0297	0.0461^**^	0.0499^**^	−0.0269
COS	0.1336^***^	0.1354^***^	−0.0692	0.1327^***^	0.1359^***^	−0.0727
ALL	0.5756^***^	0.5692^***^	−0.3184	0.5797^***^	0.5746^***^	−0.3321

Spearman correlation coefficients and significance levels resulting from the Mantel test are indicated. *P < 0.05; **: 0.01 < *P* < 0.001; ***P < 0.001. ALL: the matrices of all the environmental variables.

For the eight environmental drivers (elevation (ELE), slope (SLO), convexity (CON), aspect, topographic wetness index (TWI), altitude above channels (ACH), and sine (SIN) and cosine (COS) of aspect), ELE difference had a larger significant and positive effect (compared with Spearman’s r) on the beta diversity dissimilarity components, followed by SLO and CON; however, the difference of ACH had no significant impacts on the beta diversity dissimilarity components (with a few exceptions, e.g., β_BC.GRA_ in 2011 and β_BC.GRA_ in 2016) (Table 4). Similarly, the environmental drivers had nearly identical effects on the beta diversity dissimilarity components for both the 2011 and 2016 census (Tables 3, 4).

**Table 4:** Abundance based pairwise dissimilarity measures used in this study, including the Bray-Curtis dissimilarity measure and its notation, formula, and references.

	Pairwise dissimilarity measure	Notation	Formula
Sørensen group	Percentage difference (*alias* Bray-Curtis dissimilarity)	β_BC_	$B+C/2A+B+C$
		β_BC.BAL_	${\rm{\min }}(B,C)/A+\,{\rm{\min }}(B,C)$
		β_BC.GRA_	$\frac{|B-C|}{2A+B+C}\times \frac{A}{A+\,{\rm{\min }}(B,C)}$

*A* is the number of individuals of each species that exist at both *m* and *n* sites, whereas *B* and *C* are the number of individuals that are unique to the *m* and *n* sites, respectively^6,10^.

### Variation partitioning of beta diversity

**Figure 4:**
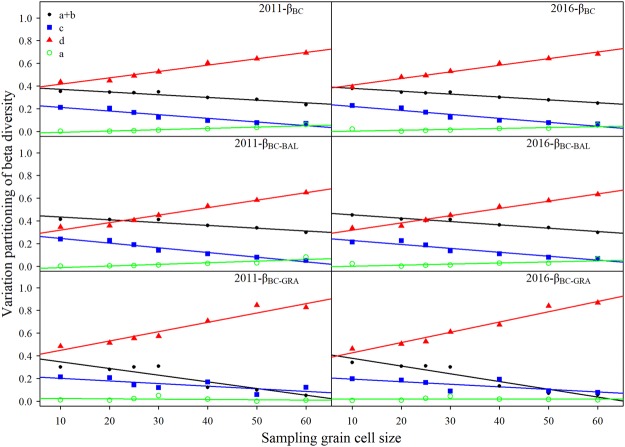
The trends of the different components of variation partitioning for the various dissimilarity components of beta diversity along different sampling grain cell sizes in the 15-ha Nnonggang plot. a + b: the pure habitat and the spatially structured habitat components that can be related to niche processes; c: the pure space that can be related to neutral processes; d: undetermined; a: pure habitat.

The variation of each dissimilarity component of beta diversity explained by the environmental component (a + b) and the pure spatial component (c) decreased systematically with the sampling grain size. We also found a marked general pattern in the undetermined variation (d), which generally increased with larger grain size. The contribution of the pure habitat component (a) had a positive relationship with the sampling grain size. However, the pure habitat effect was negligible (<8%), particularly at smaller scales (<1%). The environmental variables had a larger explanatory power than did the spatial descriptors, fitted by *dbMEM* across all sampling grain sizes with few exceptions, such as the explained abundance gradients component for the 2011 and the 2016 censuses grain sizes larger than 50 × 50 m. The fraction of undetermined variation was higher, especially at larger scales (about 80%). The pattern for the 2011 census was largely the same as for the 2016 census (Fig. 4).

## Discussion

For this study, we employed a novel abundance based approach of Baselga’s framework^6,8^ to explore beta diversity patterns, and their related components of spatial and temporal variation associated with stochastic and deterministic processes in structuring community assembly, in a 15-ha tropical karst seasonal rainforest. Our results revealed that high beta diversity could be primarily explained by a balanced variation in abundance, rather than abundance gradients. We highlighted that topographic environmental drivers contributed significantly to the maintenance of beta diversity in this heterogeneous karst ecosystem.

### Temporal dynamics of beta diversity

We found obvious changes of species assemblages in certain habitats after five years. Habitats in low-lying areas, seasonally flooded depressions, peripheral depressions, and adjacent areas exhibited rapid community changes. These areas are covered by abundant lianas and epiphytes within the interlayer and overstory, and the vegetation is thoroughly a vegetation type of tropical valley rainforest. The lianas might have accelerated the effects on species regeneration dynamics. Species in these habitats, particularly the dominant species such as *Ficus hispida*, *Sterculia monosperma* and *Vitex kwangsiensis*, possess a higher birth and death rate. Conversely, species in the elevated habitats typically have lower birth and death rates, such as *Excentrodendron tonkinense*, *Boniodendron minius*, and *Diplodiscus trichosperma*. The morphology and anatomy of these species in the elevated habitats are similar to drought resistant plants. In addition, there was much higher mortality than new recruitments after five years. One possible reason is the character of the karst climax community and natural succession. The other potential cause is that the community experiences interference by natural and artificial factors. Artificial interferents may be assigned to the routine monitoring of researchers, such as litter collection, seedling monitoring, dendrometer installation, and tree growth measurements. Natural events, such as typhoons and heavy rainfall were also not excluded.

Despite changes in local species assemblages in certain habitats, the beta diversity showed no significant change between the two censuses. It may well be that the overall abundance of species populations in each cell had no obvious change after five years. Meteorological factors over the last five years might have ben altered more than that a decade earlier; however, the colonization and localized extinction of species would not occur over such a brief timeline. Therefore, local and short-term changes in species composition may not have caused the overall total pairwise inter-site dissimilarity, which remained relatively constant between 2011 and 2016.

### Spatial dynamics of beta diversity along sampling grain cell size

A unique feature of this study site is that the high beta diversity can be primarily explained by a highly balanced variation in abundance rather than via abundance gradients. This means that the compositional dissimilarity is derived from changes in species abundance from site to site, with different signs for different species. Further, for the most part, changes balance each other, in contrast to all species changing their abundance from one site to another^6,8^. Although there was stronger topographic structuring in karst forests, variations in assemblage composition in terms of species abundance showed balanced variation patterns between different grain cells.

As a result of pooling information from smaller to larger cells, the dissimilarities of species assemblages decreased systematically with increased sampling grain cell size. This result was consistent with the expectation that if the spatial extent is fixed and sampling grain is allowed to vary, then beta diversity will decrease monotonically^26,27^. Species spatial replacement, or beta diversity induces a deterioration of the similarity in species composition with geographic distance, known as the distance-decay relationship, which typically describes how the similarity in species composition between two communities varies with the geographic distance that separates them^28^. This has generally been investigated across a wide range of organisms, geographic gradients, and environments that are geographically far apart^29^. Our study was conducted within limited spatial parameters, where species composition differed mostly in balanced variation in abundance of species between two grain cell samples. However, the results of the Mantel test indicated that differences in environmental drivers had positive effects on the patterns of species compositional dissimilarity among cells, which somewhat mimicked the distance-decay relationship.

### Environmental drivers and stochastic processes in the structuring of species assemblages

Environmental drivers exerted a potent influence on beta diversity, particularly for total beta diversity and its balanced variation in abundance. Almost all of the variations in beta diversity explained by environmental variables were spatially structured and mostly correlated with elevation, slope, aspect, etc. Species replacement along spatial or environmental gradients implied the simultaneous gain and loss of species due to environmental filtering, competition, and historical events^10^. This is not surprising given the strong spatial Fengcong-Depression elevation structure of the 15-ha plot. Guo, *et al*.^25^ showed that the area could be divided into eight distinct habitats, based primarily on elevation, aspect, and slope. The underlying rationale for this sorting might be attributable to marked differences in microenvironmental conditions, such as light, soil properties such as soil depth, as well as water and nutrient dispersion along the Fengcong-Depression gradient^25,30^. This species-specific study also supported the idea that niche partitioning plays critical role in the structuring of plant communities.

Environmental drivers and neutral processes jointly drove community assembly and meta-community dynamics, where their relative importance was modified with spatial scale. Both niche and neutral processes systematically decreased with larger spatial scales; however, the proportion of undetermined variation increased with more expansive spatial scales. This result was in sharp contrast to the findings of Legendre, *et al*.^31^ who reported on a broadleaved subtropical forest, where the relative importance of environmental factors increased, while the stochastic factors declined with larger sampling plots. The proportion of undetermined variation remained fairly constant between spatial scales. Inconsistent results were also presented by Punchi-Manage, *et al*.^32^ as relates to a tropical dipterocarp forest. Here, the relative importance of environmental factors increased, while the proportion of undetermined variation declined as the dimensions of the sampling plot increased; meantime, the stochastic factors remained unchanged between spatial scales. Our results were consistent with the hypothesis that environmental drivers dominate over neutral processes across all sampling grain cell sizes in this heterogeneous karst forest.

Numerous studies have claimed that environmental variables had a greater effect on beta diversity than did spatial effects in temperate forests^33^, while spatial effects explained a larger proportion of the variation in tropical forests^20^. However, all of these studies were based on a single grain size sample. Several recent studies revealed that environmental variables had a greater effect for larger grain size samples (e.g., 40 × 40 m), while spatial effects explained a larger proportion of the variation at smaller grain size samples^31,32^. Coincidentally, environmental differences had the greatest positive effects on the beta diversity for the 40 × 40 m grain size sample (Table 3). Gilbert and Lechowicz^34^ found a stronger environmental influence, relative to space, on beta diversity at local scales (0.1–3.5 km) in a temperate forest, whereas Condit, *et al*.^1^ found that a purely spatial model of community assembly predicted beta diversity at intermediate scales (e.g., 0.5–50 km) in tropical forests.

Why are there marked differences between different studies despite them following a similar method? It is possible that biogeographical differences (e.g., species pools and water-energy) played a significant role in community assembly mechanisms^2,20^. The degree of environmental heterogeneity (localized topographic gradients) might also strongly influence beta diversity^26^. We suggest that the environmental heterogeneity in our study site had a strong influence on beta diversity patterns, as we found that a large proportion of the variance remained unexplained by the measured environmental and fitted spatial variables, particularly in larger grain cell size samples. The proportion of variation unexplained by environment and space, representing an ‘error’ term, may be influenced by local stochasticity due to ecological drift^31^, regional sampling effects due to variations in the sizes of species pools^26^, and unmeasured environmental and spatial variables. In our view, large grain cell sized samples should be avoided in survey designs, in that large cells may obscure the effect of cell-dedicated habitat heterogeneity. Further, it is true that broad-scale topographic characteristics may be locally important^26^. Because of the complex terrains of karst ecosystems (e.g., cliffs, and caves, and sinkholes), it is difficult to use the Kriging interpolation method to precisely simulate broad-scale topographic characteristics from a fine cell (10 × 10 m). Hence, broad-scale topographic variables may not reflect the complexity and uniqueness of habitat heterogeneity in the karst ecosystem.

When uncertainties are spatially structured and not explainable by measured environmental variables they likely overestimate stochastic impacts, while underestimating environmental influences, such as the unmeasured effect of soil properties^22,35^. For example, soil variables generated more than a two-fold increase in variation that was explained by the environment, thus reducing the quantity of variation elucidated by stochastic effects^22^. The karst area in Southern China is characterized by high edaphic heterogeneity, with contrasting local-scale mosaics of soil types derived from bedrock of differing lithology. The foremost soil types are black rendzina at the summits, brown rendzina in the hillsides, and hydrated brown rendzina in the depressions, respectively. The stoichiometric characteristics of soils, such as C, N, and P content, have significant relationships with altitude^30^. The physicochemical properties of soils are the most important environmental factors missing in our analysis. However, logistical challenges in soil samples from karst terrains, particularly in the upper habitats, consisting of widespread exposed limestone surfaces and sparsely depleted soils, made it difficult to obtain data on the physicochemical properties of the soil^30^.

It is possible that we somewhat underestimated the environmental effects. A determination of whether large unexplained variations are caused by the lack of relevant data, or simply an implicit feature of the karst ecosystem remains to be elucidated. Additional unmeasured deterministic factors, such as the physicochemical properties of soil, the spatial distribution regularity of rock, or fracture distribution, might be driving the observed patterns. However, there is no doubt that abundance based dissimilarities in species composition between two assemblages is the consequence of deterministic processes, in which species abundance is variable at specific sites in a particular way in heterogeneous karst forests. Overall, in this seasonal tropical karst rainforest, deterministic processes induced by topography plays an critical role in the maintenance of species diversity due to the uniqueness of the ecosystem.

Furthermore, the relative degree to which community dynamics are deterministic or stochastic is impossible to quantify from the temporal changes in species composition alone^36^. Compared to their functional and phylogenetic composition, the formal nomenclature of species cannot convey critical information regarding their ecological and evolutionary similarity^37^. For robust inferences to be made it is critical that the analyses of species turnover go beyond the traditional approach of investigating this aspect by incorporating information pertaining to their ecological and evolutionary similarities^36^. The acquisition of additional phylogenetic and functional data facilitates the parsing of deterministic influences of different ecological filters. Hence, without further data on the phylogenetic patterns of plant or trait diversity it may be arbitrary to be so definitive. For future research, the prediction of temporal changes in species composition in natural forests may therefore be tractable only through the use of a functional trait based approach.

## Materials and Methods

### Study site and data collection

**Figure 5:**
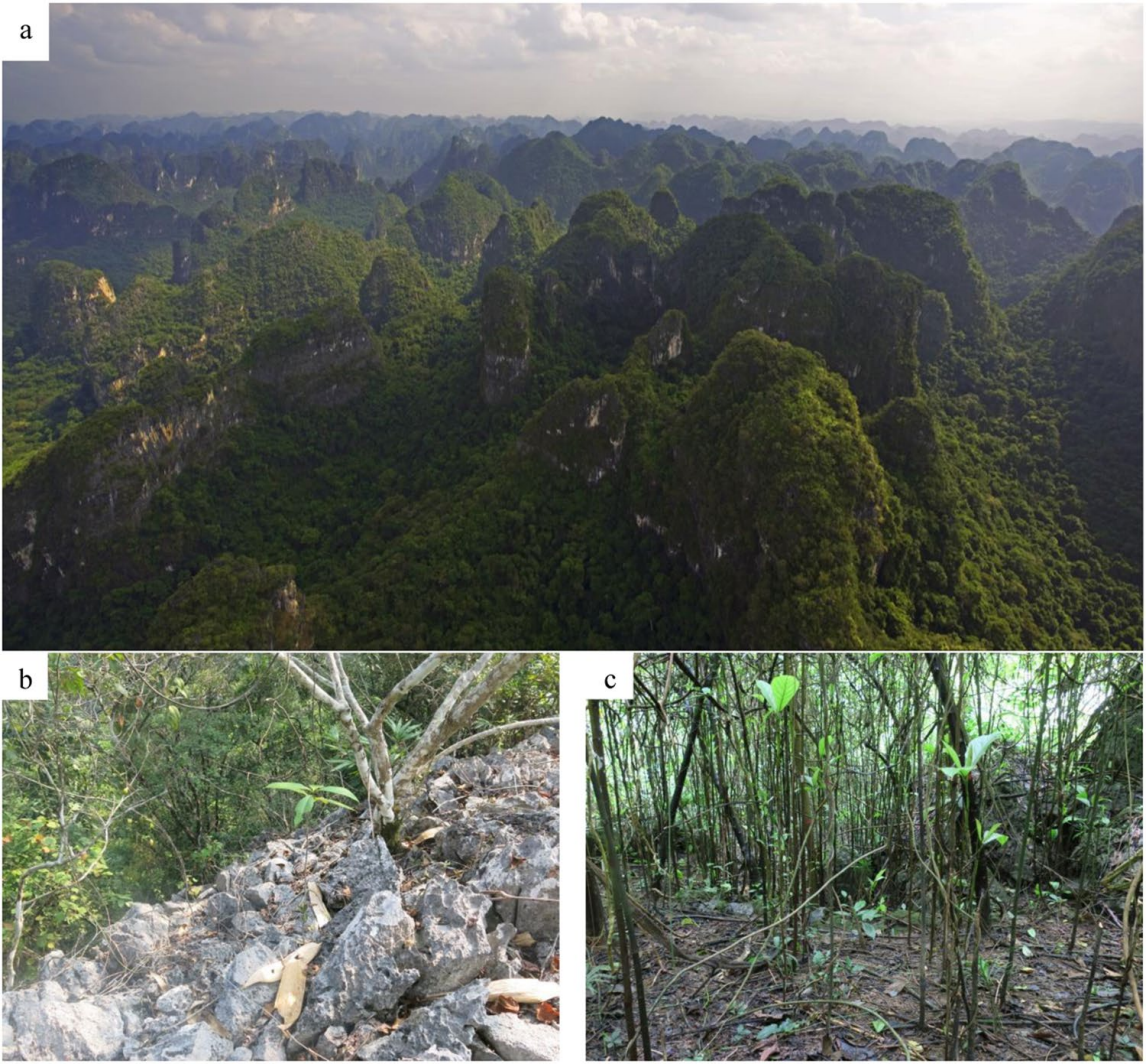
Photograph of the macroscopic community physiognomy of the tropical karst seasonal rainforest seen from a hilltop in the Nonggang National Nature Reserve in Southern China (**a**), and the microenvironmental conditions beneath the forest canopy for the hilltops of the Fengcong (**b**) and the low-lying depression (**c**).

**Figure 6:**
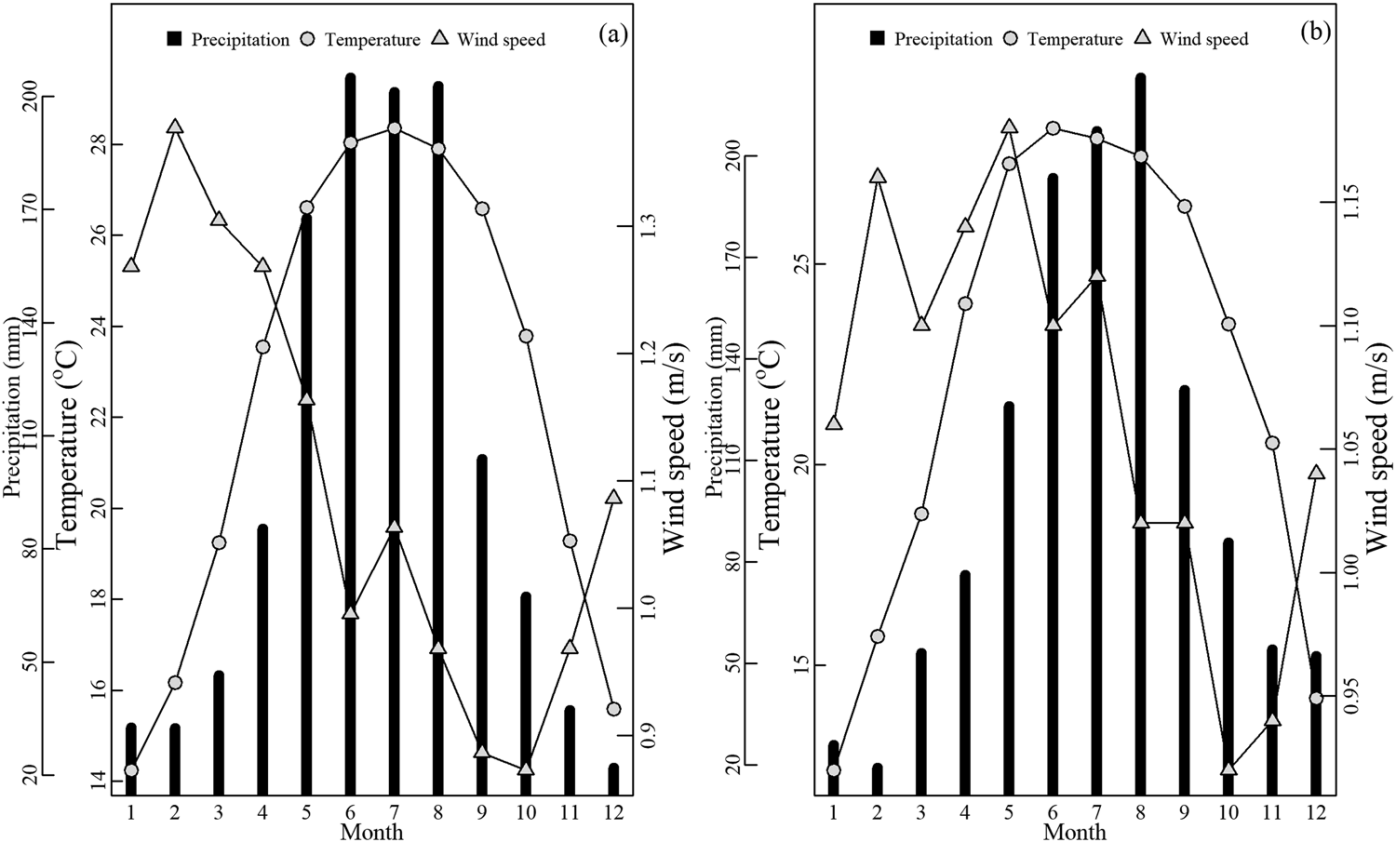
Monthly mean variations of precipitation, air temperature, and wind speed between 1971 and 2010 (**a**), and between 2011 and 2015 (**b**) in the Nonggang National Nature Reserve in Southern China.

The study site was in the Nonggang National Natural Reserve (22°13′56″–22°33′09″N, 106°42′28″–107°04′54″E), Guangxi Zhuang Autonomous Region, in Southern of China. This forest has not been subjected to anthropogenic disturbance for over one hundred years; hence, the reserve preserves the most typical and aboriginal karst seasonal rainforest of China, and even globally. Currently, the forest has attained the stable cryptogenic climax stage. Topographically, the area is characterized by a typical karst fengcong depression, which comprises a combination of clustered peaks with a common base and funneled landscapes, with altitudes ranging from 150 to 600 m (Fig. 5). The unique landforms, such as peak clusters, peak forests, low-lying land, and funnels, cause a significant variation in the availability of light, as well as the thickness and wettability of soils. Other micro-relief characteristics, such as stone trench, stone facing, and swallet, formed by abundant rock outcroppings influence small-scale habitat heterogeneity. The mean annual temperature of the reserve is 22 °C, with a daily maximum temperature that ranges from 37–39 °C, and mean minimum temperature of 13 °C. The annual precipitation, most of which occurs between May and September (Fig. 6), ranges from 1150–l550 mm; however, it can attain 2043 mm, as calculated from 40 years of data (1970 to 2010).

A 15 ha (500 × 300 m) plot (22°25′N, 106°57′E) was established in the Nonggang reserve in September 2011. This plot is very rugged, with altitudes that vary from 180 to 370 m above sea level (Fig. 1), with 10-m cell slopes that varied from 3.7 to 78.9°. All woody stems with a diameter at breast height (DBH) of ≥1 cm in the plot were mapped, measured, identified to species, and tagged following standard field procedures of the Center For Tropical Forest Science^38^. To date, this is the largest long-term monitoring of a forest plot in tropical karst worldwide. The plot encompasses one fengcong and one depression, which is typical in seasonal karst rainforests. There were 68,010 free standing individuals belonging to 56 families, 157 genera, and 223 species in the 2011 census. More details on the study plot and its floristic structure may be found in Guo, *et al*.^25^.

The second census was carried out in the summer of 2016. It required approximately two months for a field team of 30 scientists, undergraduate students, and workmen to map and record all of the data. New plants with a DBH of ≥1 cm in the plot were identified to species, enumerated, measured, and mapped following the standard of the CFTS in the second census.

### Statistical analyses

#### Beta diversity partitioning

Despite some criticism^11,13,39^, Baselga’s^5^ approach has been successfully implemented to account for spatial^14,21^ and temporal effects on community composition^40,41^. Hence, it remains an important methodological framework for beta diversity analyses. For this study, we aimed to conduct a comparative analysis using Baselga’s abundance-based metrics^6,8^ to assess spatially and temporally partitioned beta diversity patterns and their potential mechanisms. We disentangled overall pairwise beta diversity (β_BC_) into balanced variation (β_BC.BAL_) and abundance gradient (β_BC.GRA_) components using the Bray-Curtis index (Table 4), and employed the package *betapart*^42^ in R 3.4.2^43^ for these analyses.

#### Environmental drivers

We divided the 15-ha plot into 1500 10 m × 10 m quadrats and obtained accurate altitude data at each 10-metre point for all stations established in 2011. We considered six topographic variables: elevation (ELE), slope (SLO), convexity (CON), aspect, topographic wetness index (TWI), and altitude above channels (ACH) for each quadrat. Details of the calculation of the topographic variables can be found in Punchi-Manage *et al*.^32^ and Guo *et al*.^25^. Since aspect is a circular variable, we transformed the aspect data by sine (SIN) and cosine (COS) to make it linear in a linear model^44^. Furthermore, we conducted a survey of rock bareness (exposed rock on the ground with no soil, RBR) in the large plot. Further details on the survey method may be found in Guo *et al*.^24^.

To explore potential mechanisms that might explain beta diversity patterns, we performed Mantel tests^7^ with 9,999 permutations to assess the correlations (Spearman’s method) between three pairwise dissimilarity matrices, the matrices of all environmental drivers, and each of the environmental drivers for each sampling grain cell size. The environmental differences between pairwise quadrats were standardized by the Euclidean Distance as the dissimilarity metric. Mantel tests were computed using the *mantel* function in the *vegan* package^45^ in R 3.4.2^43^.

#### Variation partitioning

Variation partitioning analysis of community composition across environmental and spatial gradients provide insights into mechanisms underlying community assembly. Following the recommendation of Legendre *et al*.^44^, we used the third-degree polynomial function of six topographic variables: ELE, SLO, CON, TWI, ACH, and RBR (yielding 18 variables), including two aspect derivatives (SIN and COS). Thus, we obtained 20 reconstructed environmental variables from the seven original topographic variables for variation partitioning analysis.

As spatial descriptors, we used distance based Moran’s eigenvector maps (*dbMEN*), formally known as Principal coordinates of neighbor matrices (PCNM), which were derived from the spectral decomposition of the spatial relationships between among grid cells^46,47^. This technique produces linearly independent spatial variables that cover a wide range of spatial scales, which enables the modeling of any type of spatial structure^48^. The truncation distance was selected to retain links between neighboring horizontal, vertical, and diagonal cells. We used the default for the truncation distance in the analyses. The default is to use the longest distance to keep data connected. The distances above truncation threshold are given an arbitrary value of four times threshold^48^. All eigenvectors associated with Moran’s *I* coefficients larger than the expected values of *I* were retained in the analysis, as the spatial variables for variation partitioning analysis.

We employed eigenfunctions (with only positive eigenvalues) of *dbMEM* as explanatory variables to represent spatial structure^48^, and the 20 topographic habitat variables described above to represent the environment. We used the forward selection method to extract the significant eigenfunctions of *dbMEM* and habitat variables from the above. This was done by permutation tests with 9,999 randomizations^49^. Subsequently, we used the response variable together with the two sets of variables (i.e., *dbMEM* and environmental variables from the forward selection) in the variation partitioning to determine the individual and joint contribution of *dbMEM*, and environmental variables to describe the species beta diversity^50–52^.

Variation partitioning was utilized to assess the amount of variation in the three pairwise dissimilarity matrices explained by four components: (a) pure habitat, (b) spatially structured habitat, (c) pure space, and (d) undetermined. The proportion of variation explained by the pure habitat and the spatially structured habitat components (a + b) could be related to niche processes, while the pure space (c) can be related to neutral processes^44^. However, the pure space component (c), may be attributed to a mixture of factors, including the contributions of unobserved and spatially structured environmental variables and spatial structuring processes of community dynamics^31,48^.

For the Bray-Curtis index, the dissimilarity matrices D are not Euclidean; however, ${D}^{(0.5)}=[{D}_{hi}^{0.5}]$ are Euclidean^7,10^. The square root of the dissimilarities may be used in the following distance based redundancy analysis of the variation partitioning. The *pcnm* function in the *PCNM* package^53^ was employed to create the spatial variables, and the *forward.sel* function in the *packfor* package^49^ was used to perform the forward selection. Variation partitioning analysis was computed using the *varpart* function in the *vegan* package^45^.

To determine the scaling properties of the habitat topographic driven species assemblages, we calculated eight topographic variables for 10 × 10 m, 20 × 20 m, 25 × 25 m, and 50 × 50 m quadrates at 10-metre resolution following the method described above, which allowed a division of the overall plot into cells of equal sizes. Further, for the 30 × 30 m and 60 × 60 m (for 480 × 300 m), and 40 × 40 m (for 480 × 280 m) cells, we selected only a portion of the 15-ha plot, and discarded the margin, which was not sufficient for specific cells. There were up to 1,124,250 (${C}_{1500}^{2}$) pairwise inter-site values for each component of beta diversity for the 10 × 10 m cell. Therefore, this study focused primarily on the results of the 20 × 20 m cell analysis, with 70,125 (${C}_{375}^{2}$) pairwise inter-site values.
